# Encoding location and serial order in auditory working memory: evidence for separable processes

**DOI:** 10.1007/s10339-012-0442-3

**Published:** 2012-05-23

**Authors:** Franco Delogu, Tanja C. W. Nijboer, Albert Postma

**Affiliations:** 1 Department of Experimental Psychology, Helmholtz Institute, Utrecht University, Heidelberglaan 2, 3584 CS Utrecht, The Netherlands; 2 Department of Neurology, University Medical Centre, Utrecht, The Netherlands; 3 Rudolf Magnus Institute of Neuroscience and Center of Excellence for Rehabilitation Medicine, University Medical Center Utrecht and Rehabilitation Center De Hoogstraat, Utrecht, The Netherlands

**Keywords:** Automatic encoding, Attention, Spatial, Serial order, Auditory working memory, Environmental sounds

## Abstract

In this study, we investigated the interactions between temporal and spatial information in auditory working memory. In two experiments, participants were presented with sequences of sounds originating from different locations in space and were then asked to recall either their position or their serial order. In Experiment 1, attention during encoding was manipulated by contrasting ‘pure’ blocks (i.e., location-only or serial-order-only trials) to ‘mixed’ blocks (i.e., different percentages of spatial and serial-order trials). In Experiment 2, ‘pure’ blocks were contrasted to blocks in which spatial and serial-order trials were intermixed with a third task requiring a semantic categorization of sounds. Results from both experiments showed that, whereas serial-order recall is linearly affected by the simultaneous encoding of a concurrent feature, the recall of position is mostly unaffected by concurrent feature encoding. Contrastingly, overall performance level was lower for spatial recall than serial recall. We concluded that serial order and location of items appear to be independently encoded in auditory working memory. Serial order is easier to recall, but strongly affected by the processing of concurrent item dimensions, while item location is more difficult to recall, but relatively automatic, as shown by its strong resistance to interfering dimensions in encoding.

## Introduction

When keeping track of events in memory, we have to remember *what* happened, *where* it happened, and *when* it happened. Do we maintain these different dimensions of the stimuli in integrated representation in working memory or do we have separate traces for each one of these different domains?

On the one side, numerous studies have indicated that information about the identity, the location, and the serial order of perceptual objects can be independently encoded in working memory (WM). For example, evidence of a separate encoding for objects and locations in visuospatial WM has been found repeatedly (e.g., Klauer et al. 2004, see also Zimmer 2008 for a review). Also, serial order *per se* appears to be dissociable from the type of information contained in the item sequence (Amiez and Petrides 2007; Kesner et al. 1994; Milner et al. 1991) and from the location of items within the sequence (Dutta and Nairne 2003; Healy 1975). Moreover, recent neuroimaging studies confirmed that the encoding of item identity, location, and serial order seems to be mediated by different brain regions (see Courtney et al. 2007 for a review).

On the other side, several studies have shown that these event features can be integrated into unified memory representations through mechanisms of feature binding (see, e.g., Prabhakaran et al. 2000; Jiang et al. 2000; Maybery et al. 2009). Accordingly, Baddeley’s revised working memory model included the episodic buffer as a component, which is responsible for integrating different information in short-time multidimensional representations (Baddeley 2000).

Until recently, however, not all binding processes have been studied in depth. While feature–feature binding (see the seminal works of Treisman 1999 and Luck and Vogel 1997) and feature–location binding (see among others Prabhakaran et al. 2000) have been extensively investigated, less attention has been devoted to exploring the mechanisms of binding between serial order and location of items. A remarkable exception is offered by a recent study by Gmeindl et al. (2011), which focused on how serial-order information is associated in encoding to either item identity or item location. The authors compared memory span tasks for locations and letters in conditions in which serial order was either task-relevant or task-irrelevant. Participants failed to detect changes in serial order more in the spatial task than in the identity task. Moreover, when participants were not required to remember serial order, they tended to recall the correct serial order for item identity, but not for item location. They argued that the maintenance of verbal identity and spatial information is achieved through different rehearsal mechanisms, a serial rehearsal for verbal information and a multilocation configural rehearsal for spatial information. The authors concluded that serial order is more efficiently bound to the identity of the stimuli than to specific spatial positions. By contrast, there are studies indicating that verbal and spatial stimuli show similar or functionally equivalent serial position curves (Smyth and Scholey 1996, see also Parmentier 2011 for a review). Such equivalence suggests that, analogously to verbal encoding, the spatial encoding of a sequence of locations does involve serial processing.

Cross-domain interference between spatial and temporal features has been investigated by Dutta and Nairne (2003). Their participants selectively attended either to spatial or to temporal (serial) information during a speeded classification task while ignoring irrelevant variation along the other dimension. They found that whereas participants can selectively ignore temporal or spatial variation when no recall of the irrelevant dimension is required, they suffer interference when information from both dimensions must be remembered (Dutta and Nairne 2003). Similar results have been found by van Asselen et al. (2006). They asked participants to recall either the serial order or the exact individual positions of sequentially presented visual items. In order to investigate the automaticity of spatiotemporal integration, they manipulated attention toward each one of the two dimensions by biasing the expectancy of attending either to a spatial or to a temporal task across different blocks of trials. In two ‘pure’ blocks, participants were exclusively presented with temporal or spatial trials. In two ‘mixed’ blocks, they were presented with the majority of trials (80 %) within one dimension (temporal or spatial) and the remaining trials (20 %) within the alternative dimension. Results showed higher accuracy in expected tasks than in the less-expected task both in the spatial and the temporal domains. The authors concluded that attention plays an important role during the encoding of both the location and the serial order of visual objects (van Asselen et al. 2006). Performance in the 20 % condition was clearly above chance, though, suggesting that there is also partial automatic encoding of the unattended feature.

Taken together, the above-mentioned studies offer a first indication that an integration of serial and spatial information of items, with or without the simultaneous encoding of item identity, is a markedly demanding process. In fact, it seems that spatial–temporal binding is more difficult than identity–temporal binding (Gmeindl et al. 2011) and that the simultaneous maintenance of serial order and location of items is an effortful process (Dutta and Nairne 2003), which is significantly modulated by the distribution of attention resources toward the two dimensions during encoding (van Asselen et al. 2006).

Since these previous studies have been conducted with visual stimuli only, it is unknown whether such location–order binding costs could be generalized to other sensory modalities. A comparison to the auditory domain is particularly relevant, as there is strong evidence indicating vision being dominant in spatial processing (Kubovy 1988; Morein Zamir et al. 2003), whereas audition is dominant in temporal and sequential processing (Kubovy 1988; Conway et al. 2009). Accordingly, it is possible that such a primacy of temporal processing over spatial processing in the auditory domain could affect location–order binding.

In line with the foregoing, in the current study, we investigated how spatial and temporal information are combined with each other in auditory working memory encoding. We modified the experimental paradigm used in van Asselen’s study (2006), adapting it to the auditory modality and adding new experimental conditions. We presented participants with two blocks of trials that were either exclusively temporal or exclusively spatial, as well as with two blocks of trials in which the majority of trials (80 %) was within one domain (temporal or spatial) and a minority of trials (20 %) was within the other domain (spatial or temporal; hence, less expected). Most importantly, we added a fifth block in which the expectations of recalling the spatial and the temporal dimensions were equal (50–50 %). As participants did not know which of the two alternative tasks they were going to perform, they were forced to encode and to maintain in memory all types of information, the serial order, the spatial location, and obviously, the identity of the auditory items.

We hypothesized that if the location and serial order of auditory items were automatically integrated in a joint representation in auditory WM, no differences in their recall should be found as a function of the amount of attention dedicated to the target dimension during encoding. On the contrary, if the two dimensions were not automatically integrated, the intention to learn should play a significant role, and accuracy should increase as a function of the amount of attention devoted to the target feature.

Moreover, it is possible that one of the two features, either the position or the serial order of items, is more primarily and more automatically encoded. In this case, we expect dual encoding to have less negative effects on the recall of the primary feature. We tested these hypotheses in two experiments. In the first experiment, participants were expecting to recall either the items’ location only or the items’ serial order only or both dimensions. In the second experiment, we contrasted trials where they expected to recall only location or serial order with trials requiring also the encoding of a non-spatial, non-temporal stimulus dimension.

## Experiment 1

In this first experiment, we aimed at testing whether information about item location and item serial order are automatically integrated in auditory WM encoding. Sequences of auditory stimuli were presented from five different locations, and participants were asked to recall either their location or their serial order. Attention was manipulated by varying the proportion of serial-order and spatial trials between blocks (i.e., 100, 80, 50, and 20 % of location versus serial-order trials).

## Method

### Participants

Twenty students of the University of Utrecht (mean age: 25.5 years (SD: 5.9), 11 females) participated in exchange for course credits or a small amount of money. All participants reported normal hearing and they were all right-handed.

### Apparatus

**Fig. 1:**
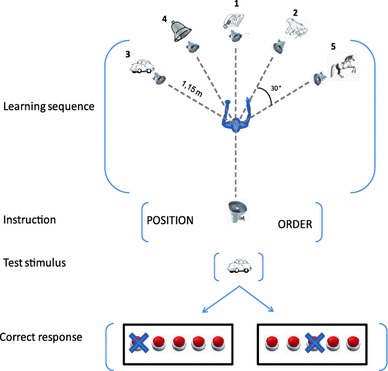
Set-up of the six loudspeakers (five for stimuli presentation and one test loudspeaker, located behind the participant) and example of a learning–test sequence. The *numbers* indicate the order of presentation. The crosses on the response *keys* indicate the correct answer for the two conditions

Five loudspeakers were used to present the auditory sequences (see Fig. 1). They were positioned 30° apart from each other in azimuth, at angles of −60°, −30°, 0°, +30°, and +60° (0° corresponds to the position faced by the participant). The loudspeakers were placed at about the head height of the seated participant (1.25 m above the ground), at a distance of 1 m from the participant’s head. A sixth loudspeaker (hereafter *test loudspeaker*) was positioned behind the participant (180° angle), at the same height as the other five speakers and approximately 60 cm behind the participant’s head. Sound-absorbing curtains were arranged on the wall in order to minimize sound wave reflection. All sounds were presented with an average loudness of 70 dB. A response box was placed in front of the participant. The position of the keys on the response box was arranged in an ergonomic way in order to reduce muscular tension and fatigue. An 8-channel audio card controlled by a custom-written Matlab (The Mathworks, MA) script was used for the presentation of the sounds through the test loudspeakers.

### Stimuli and tasks

The stimuli consisted of a set of 40 environmental sounds, described in a previous study (see Delogu et al. 2009). Environmental sounds instead of spoken words were chosen to allow for a more direct comparison of this study to previous studies that also used non-linguistic stimuli (pictures) in the visual domain (see, e.g., Van Asselen et al. 2006). All sounds lasted 2 s. All stimuli belonged to one of the three following semantic categories: human sounds (e.g., baby crying, person coughing), animal sounds (e.g., cat meowing, bird chirping), and tool sounds (e.g., car engine, telephone ring). The sounds were presented in sequences of five sounds, each of them originating from a different loudspeaker. All the sequences contained a random selection of items, with the limitation that a sound could not be presented in a sequence if it was already presented in one of the previous two sequences. The experiment included five different blocks of trials: two blocks of 10 sequences each (‘pure’ blocks), in which participants had to perform only one task (either the location or the serial-order task) throughout the entire block; two blocks of 20 sequences each (80–20 % blocks), in which participants had to recall one feature in the 80 % of the trials and the alternative feature in the remaining 20 %; and one block of 16 sequences (50–50 % block), in which the participants were requested to perform the location task in the 50 % of trials and the serial-order judgment in the remaining 50 %. The order of presentation of the five blocks of trials was counterbalanced between participants.

### Procedure

Participants were first trained to use the five keys to indicate either the position of the sound (with the leftmost key indicating the leftmost loudspeaker and the rightmost key indicating the rightmost loudspeaker) or to indicate its serial order (with the leftmost key corresponding to the first sound in the sequence and the rightmost key corresponding to the last sound in the sequence). Before starting the experiment, they also performed an auditory localization task in which they were asked to indicate the position of a series of 100 sounds randomly originating from one of the five speakers. Results of the sound localization task showed high accuracy (mean: 90 %, SD: 4 %), indicating that the azimuthal separation between auditory sources was easy to detect. Since the smallest detectable change in angular position in azimuth is always lower than 4° for the positions included in our task (Mills 1958), we were safely above sensory threshold levels. The separation of 30° was chosen as a compromise between two contrasting needs: (1) to have a sufficient discriminability between speakers and (2) to reduce the use of easy categorical labels to encode item positions (e.g., cardinal points with 45°).

During the experiment, detailed auditory instructions indicating which task the participants were about to perform (serial order versus location) were presented before each block. In the two 80–20 % blocks, participants were explicitly told which feature they would be asked to recall in the majority of the trials. They were also told that in a marginal amount of trials, they would be asked to perform the alternative task. In the 50–50 % block, participants were explicitly told that no feature was prevalent and that, in order to increase their chances of a correct recall, they should pay attention to both features during the sequence presentation. The manipulation of the probability of the task (100, 80, 50, and 20 %) was explicit and unequivocally explained to the participants in order to bias their attention toward either the spatial or the serial-order features during encoding. In such a way, for each tasks, we operatively defined the following four conditions of attention: full attention (100 %), partially diverted attention (80 %), divided attention (50 %), and marginal attention (20 %).

Participants triggered the presentation of each learning sequence by pressing a key on the response box and then listened to the five sounds. After listening to the learning sequence, they were presented with the instruction word (either ‘ORDER’ or ‘POSITION’) coming from the test loudspeaker, indicating which feature they had to recall. Then, all the sounds of the learning sequence were presented again, one by one in a random order, from the test loudspeaker. After each test sound, participants had to recall, according to the condition, either which location or which serial order that sound had in the learning sequence (see Fig. 1). The experiment lasted approximately 70 min. The procedure we employed to assess the memory of the serial order differs substantially from traditional methods such as the immediate serial recall (ISR) task. The reason of using alternative measures is due to the exigency to integrate the serial-order task with the object location memory task in different attentional conditions.

### Analysis

A two-way repeated measures ANOVA with the variables *feature* (location vs. serial order) and *expectancy* (100, 80, 50, and 20 %) was performed on the mean percentage of correct responses. The Greenhouse–Geisser correction was applied whenever the assumption of sphericity was violated. Fisher’s LSD was used for post hoc comparison. Two participants were excluded from final analysis because their performance, at least in one condition, was more than 2 standard deviations under the group average. *F* (2, 21) = 13.74.

## Results

A main effect was found for *feature*, *F* (1, 19) = 11.73, *p* = 0.003, $\eta_{p}^{2}$ = 0.382, showing that serial order of items was easier to recall than items’ location. *Expectancy* also yielded a main effect, *F* (3, 57) = 16.89, *p* < 0.001, $\eta_{p}^{2}$ = 0.47, indicating that the accuracy in the recall of one of the two features increases together with the expectation of recalling such feature.

**Fig. 2:**
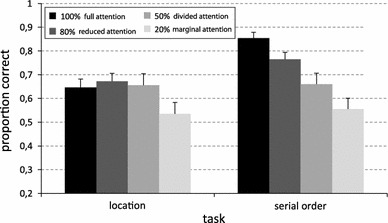
Accuracy in the location and in the serial-order tasks split on level of expectancy (100, 80, 50, and 20 %). *Error bars* represent standard errors from the mean

Importantly, a significant interaction between *feature* and *expectancy* was obtained, *F* (3, 57) = 5.21, *p* = 0.003, $\eta_{p}^{2}$ = 0.215. Taking a closer look at this interaction (see Fig. 2), it can be observed that task expectancy strongly and linearly influenced the accuracy in the serial-order task, while affecting the location task only marginally. Post hoc pairwise comparison showed that accuracy in the 100 % location condition, where participants only had to memorize the location of sounds, was equivalent to the accuracy in the 80 % location condition, where the attention in encoding was partially diverted toward serial order (*p* = 0.434), and also to the 50 % location condition, where participants were instructed to encode both features (*p* = 0.922). The only condition in which spatial recall was significantly impaired was the 20 % condition, in which location recall was strongly unexpected (*p* = 0.041 in the 100 vs. 20 % comparison).

On the contrary, in the serial-order task, accuracy exhibited a linear effect of expectancy: the more expected was the recall of serial order during encoding, the better the serial recall. Post hoc tests (*p* < 0.05 in all pairwise comparison between serial-order conditions) showed that accuracy was progressively poorer as the probability of performing the alternative task increased. It is worth to report that the accuracy in the 20 % condition was higher than chance level. In fact, two separated one-sample *t* tests indicated that the accuracy in the location and in the serial-order tasks were both significantly different from the chance level: *t* (19) = 5.8, *p* < 0.001 for the location task and *t* (19) = 6.6, *p* < 0.001 for the serial-order task.

**Fig. 3:**
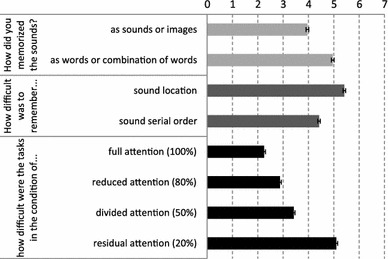
Post-experimental questionnaire. *Error bars* represent standard errors from the mean

A post-experimental questionnaire was administered to all subjects in order to provide descriptive statistics about the strategies used to maintain items, the perceived difficulty of the location and serial-order tasks, and to verify the influence of attention in their subjective perception of the difficulty of the tasks. Results are summarized in Fig. 3.

## Discussion Experiment 1

In Experiment 1, we investigated whether location and serial-order information are integrated in working memory. We compared recall accuracy in blocks of trials in which participants were asked to attend only one dimension (full attention blocks) to accuracy in blocks in which the recall of one of the two dimensions was prevalent (80–20 % blocks) and to accuracy in blocks of trials in which participants had to attend to both features (divided attention blocks).

Accuracy in the full attention blocks was markedly higher in the temporal task (86 %) than in the spatial task (65 %). This result appears to support the notion that auditory modality is specifically tuned to the processing of serial-order information. Alternatively, it could be due to differences in spatial and serial-order rehearsal. In fact, while serial-order maintenance can be achieved through the rehearsal of the mere item identity, which is likely to be serial even when not required (cf. Gmeindl et al. 2011), the maintenance of item location needs additional, specifically spatial processing. A final possibility is that the crucial factor here is the low accuracy in the location task, which reflects the general lower sensitivity of the auditory modality for space. It should be mentioned here, though, that in the discrimination trials reported in the method section, we observed that perceptual discrimination of the sound location was quite high for the present set-up. Notably, van Asselen et al. (2006) observed a higher performance (80 %) in a visual location task than what we found in the auditory location task (65 %), although they used larger (7 items) stimuli sequences. Admittedly, the present experimental set-up differed in more dimensions from previous studies than only in the stimulus modality.

Concerning the overall influence of attention, our data indicated that participants were more accurate when expecting to recall only one feature instead of both. This result suggests that, in auditory WM, an integrated representation of serial-order and spatial information is not automatic. This result is consistent with studies by Dutta and Nairne (2003) and by van Asselen et al. (2006) in the visual domain. The post-experimental questionnaire revealed that the subjective perception of the difficulty of the task progressively increases when attention is reduced.

More interestingly, our results indicate that the attention during encoding affects the serial-order task more than the location task. Specifically, item location memory appears less affected by expectancy than serial-order memory. As tolerance toward concurrent processing loads is a sign of automaticity (Andrade and Meudell 1993; Ellis 1990), we argue that spatial encoding is more automatic than serial-order encoding in the auditory domain. This result is consistent with previous findings in the visual domain, where spatial information has been demonstrated to be relatively automatically processed even when attention is focused on other features (Köhler et al. 2001; Olson and Marshuetz 2005). It is worth noting that this last result is not consistent with the expected primacy of temporal and sequential processing over spatial processing in the auditory domain (Kubovy 1988; Conway et al. 2009).

From the results of Experiment 1, we cannot decisively explain why dual encoding does not impair sound localization. It could either be that the specific kind of concurrent information (i.e., serial order) does not interfere with spatial processing, or that the encoding of sound position is resistant to the interference of concurrent information, no matter if it is serial or not. Similarly, we cannot decide whether serial-order encoding is specifically affected by concurrent spatial information or whether it would also be affected by non-spatial information. In order to verify if such non-mutual effects of attention on spatial and serial-order recall are dependent or independent from the type of concurrent information, we conducted a second experiment in which spatial and serial order were respectively combined with the encoding of a non-spatial and non-serial feature of the stimulus.

## Experiment 2

In Experiment 1 information about serial order and item location was used both as target feature and as interfering memory load. Consequently, it is difficult to establish why spatial encoding was not impaired by the concurrent encoding of serial-order information. In Experiment 2, location and serial-order information had to be encoded either alone or while participants had to remember a third, independent feature. The new feature to be encoded was the semantic category of the environmental sounds, which could belong either to the group of living things (i.e., human or animal sounds) or to the group of non-living things (i.e., tool sounds). We hypothesized that if even when combined with new concurrent information, spatial encoding was less interfered by a concurrent memory load than serial-order encoding, we could conclude that spatial encoding is more automatically encoded than serial order in auditory WM.

## Method

### Participants

Twelve students of the University of Utrecht (mean age: 25.5 (SD: 4.4), 8 females) participated in exchange for course credits or a small amount of money. All participants reported normal hearing and they were all right-handed. None of the participants who took part in Experiment 1 have also participated in Experiment 2.

### Apparatus

Same as in Experiment 1.

### Stimuli and tasks

The stimuli were the same as in Experiment 1 and they were presented in sequences of five items like in Experiment 1. A new task (*semantic categorization task*) was added in which participants were asked to memorize if each one of the sounds of the sequence was produced by a living thing, like an animal or a human being, or by a non-living thing, like a machine or a tool. The task required to indicate in the test phase how many living things were presented in the sequence. Additionally, in order to force participants to encode stimulus category during the sequence and to prevent post-encoding response strategies, we asked participants to press a pedal with their right foot during sequence presentation every time that a sound was produced by a living thing. The experiment included five different blocks of trials: three blocks of 10 sequences each (‘pure’ blocks), in which participants had to perform always the same task (either the location, the serial order, or the categorization task) throughout the entire block; one block of 20 sequences each (50 % location and 50 % categorization), in which participants were requested to perform the location task in the 50 % of the trials and the semantic categorization task in the remaining 50 %; one block of 20 sequences (50 % order and 50 % categorization), in which participants were requested to perform the serial-order task in the 50 % of the trials and the semantic categorization task in the remaining 50 %. In order for participants to familiarize with the categorization task, all participants started the experiment with the pure categorization block of trials, while the order of the remaining 4 blocks was pseudo-randomized between participants according to a Latin square presentation design. The pedal-press task in the presence of living stimuli was required in all the blocks of trials in which the categorization task was included.

### Procedure

The procedure was analogous to the one in Experiment 1.

### Analysis

One of the participants was excluded from final analysis because his performance was more than 2 standard deviations under the group average. A two-way repeated measures ANOVA with the variables *feature* (location versus order) and *expectancy* (100 vs. 50 %) was performed on the proportion of correct responses. Concerning the semantic categorization task, we measured accuracy as the difference between the reported number of living things and the actual number of living things. We obtained a measure ranging from 0 (no error) to 5 (all sounds wrongly categorized). The proportion of categorization errors was used as dependent variable in a separate unifactorial ANOVA with *expectancy* as factor with three levels: 100 % categorization, 50 % categorization with location concurrent encoding, and 50 % categorization with serial-order concurrent encoding. The Greenhouse–Geisser correction was applied as the assumption of sphericity was violated.

## Results

Concerning the location and the serial-order tasks analysis, a main effect was found for *feature*, *F* (1, 11) = 7.45, *p* = 0.019, $\eta_{p}^{2}$ = 0.404), showing that overall accuracy was higher in the order task (75 % correct) than in the location task (64 % correct). *Expectancy* also yielded a main effect, *F* (1, 11) = 5.75, *p* = 0.035, $\eta_{p}^{2}$ = 0.343, with higher accuracy in the expected trials than in the less-expected ones.

**Fig. 4:**
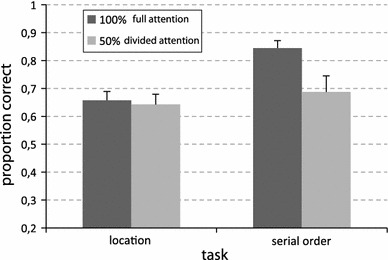
Accuracy in the location and in the serial-order tasks in the pure blocks (100 %) and in the blocks associated with semantic category encoding (50 %). *Bars* represent standard errors of the mean

The *feature*–*expectancy* interaction showed the strongest effect of the analysis: *F* (1, 11) = 13.97, *p* = 0.003, $\eta_{p}^{2}$ = 0.560. Dual encoding selectively impaired serial-order recall and it did not influence spatial recall. As shown in Fig. 4, while recalling serial order, participants were less accurate (*p* = 0.003) in the condition that also required encoding the living thing property than in the single encoding condition (only serial order). On the contrary, while recalling item location, accuracy was equivalent (*p* = 0.874) for dual encoding (both location and living thing property) and single encoding (only location).

Concerning the semantic categorization task, participants correctly responded with a pedal press to *living* stimuli after 1,518 ms in average, with a standard deviation of 504 ms. After the sequence presentation, they showed low rates of error in counting how many *living* stimuli were presented in the sequence (7 % for the pure categorization block, 2 % when mixed with the spatial task, and 4 % when mixed with serial-order task). A significant effect of *expectancy* on the proportion of errors was obtained, *F* (2, 22) = 8.58, *p* = 0.003, $\eta_{p}^{2}$ = 0.438. The direction of this effect is surprising: participants, as shown in the post hoc analysis, performed worse in the ‘pure’ living thing task condition compared to both mixed conditions (*p* < 0.05 in the pairwise comparisons with both mixed conditions). Most likely, this result is due to a learning effect, with participants improving their performance in the blocks following the first one, which was always the ‘pure’ categorization one. Moreover, performances in the two mixed blocks were not statistically different (*p* = 0.478), indicating equivalent interference of spatial and serial-order encoding on the living thing task.

## Discussion Experiment 2

The main goal of Experiment 2 was to test whether memory for sound location and serial order was affected by the concurrent encoding of the semantic category of to-be-recalled sounds. The performance in the ‘pure’ 100 % location and serial-order conditions closely resembled what we observed in Experiment 1: participants were more accurate in the serial order than in the location task. Concerning the categorization task, accuracy in all conditions was high (above 90 %), indicating that participants found it easy to categorize sounds as produced by living things and also that they paid attention to this task even when paired with the spatial or temporal concurrent tasks.

More interestingly for our theoretical purposes is that dual encoding affected more the recall of the serial order than the recall of the location of items, even when the concurrently processed feature was both non-spatial and non-serial. More specifically, when attention during encoding was divided between the location of items and their semantic categorization, performance in the location task was equivalent to when participants could focus exclusively on spatial encoding. By contrast, when participants had to divide their attention between serial order and categorization of items, their performance was significantly worse than when they only had to focus on serial-order encoding. These data seem again to suggest stronger automaticity for the encoding of spatial information in auditory modality, independently from the nature of the interference during encoding.

## General discussion

This study investigated the integration of spatial and serial-order information in auditory working memory. In two experiments, we presented sequences of five auditory stimuli from five different locations asking participants to recall either the position or the serial order of those sounds. Attentional focus during encoding was manipulated by contrasting blocks of trials containing only spatial or temporal trials to mixed blocks containing different percentages of spatial and temporal trials (Experiment 1) and by contrasting pure blocks of trials to blocks in which spatial or serial-order trials were intermixed with semantic categorization trials (Experiment 2).

In both experiments, we found an overall higher accuracy for serial order than for spatial location recall. This superior serial-order performance may be due to either a greater sensitivity of the auditory system for temporal information or a lower sensitivity for spatial information, or because the rehearsal of identity information (i.e., the sounds) takes place specifically in a serially ordered manner (e.g., Gmeindl et al. 2011). The latter option raises the question: to what extent does identity information maintenance in the current experiments depend on a verbal naming mechanism? Indeed, a rather commonly used way to memorize items is to give them a verbal label and to overtly or covertly rehearse these labels. Moreover, in the post-experimental questionnaire, our participants often reported the use of sound names as a strategy to remember items’ sequences. To establish if verbal recoding could have played a role here, an articulatory suppression (AS) condition could have been included. However, for several reasons, we decided not to include AS in the experimental design. First, since the requested tasks were already rather complex and demanding, we reasoned that an additional requirement could cause an extreme drop of accuracy. Second, we argued that the inclusion of AS could cause a selective, unbalanced impairment in the serial-order task since AS has a larger effect on serial-order than on spatial encoding (cf. Dent and Smyth 2005). Third, even if sounds were recoded into verbal labels, this would likely have happened in both the spatial and in the temporal conditions, leaving the nature of the task effects independent from verbal recoding.

**Fig. 5:**
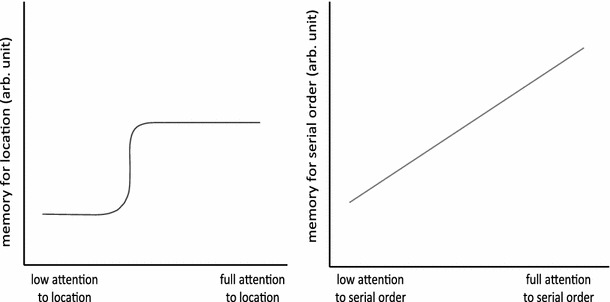
Profiles of the effects of attention on the recall of location and serial-order information

Concerning the role of attention, we found that varying the amount of attention on the target feature during encoding caused different effects in the recall of the spatial and the temporal dimensions. In both experiments, serial-order recall accuracy was linearly affected by the amount of expectancy for the serial-order feature, whereas the accuracy in the location recall was affected to a much lower extent by expectancy. In fact, spatial recall performance was significantly impaired only in the condition in which the probability of attending the location task was highly unlikely (20 %), indicating that spatial encoding is more robust than serial-order encoding for concurrent encoding of interfering information. This result suggests that, also in the auditory modality, spatial encoding is largely automatic, similarly to what has previously been found for vision (Andrade and Meudell 1993; Ellis 1990; Köhler et al. 2001). On the basis of the present findings, we may speculate that allocation of attentional resources is more crucial for temporal encoding than for spatial encoding. Both experiments show that spatial encoding is not affected by a condition requiring division of attentional resources (50 %). Experiment 1 suggests an attentional threshold model (see Fig. 5): as long as a certain amount of attention is allocated to the spatial location feature, spatial recall performance is maximal, though not optimal. Only when there is an extreme drop in attentional allocation (i.e., the marginal attention condition, 20 %), spatial recall also suffers. Differently, temporal-order encoding decreases linearly with the amount of attention spent on the temporal-order feature. The marginal attention condition (20 %), although leading to significantly impaired performances in both tasks, is anyway performed with an above-chance-level accuracy. The impairment of serial-order memory in reduced attention conditions was previously found in the visual domain (Van Asselen et al. 2006). Such comparable attentional effects in the auditory and visual modalities during serial-order tasks are likely due to the presence of supra-modal mechanisms in serial processing (Depoorter and Vandierendonck 2009). Moreover, we recently conducted a study to compare the effects of attention on spatial and serial-order encoding in visual and auditory working WM (Delogu et al. 2012). The findings of our cross-modal study, conducted with a similar experimental procedure of this present study, confirm that spatial encoding is more automatic than serial-order encoding in both the visual and the auditory domains and it suggest the presence of supra-modal mechanisms in different domains of working memory.

It is difficult to further explain why temporal order and spatial processing would diverge in their dependence on attentional control. We may speculate that such difference has an evolutionary explanation. In fact, the automaticity of the ability to encode and remember the location of items in our surroundings has been more critical for survival throughout the evolutionary history (e.g., in localizing threats and food sources) than the encoding and remembering of the serial order of items. Moreover, the greater automaticity of spatial encoding could depend on different rehearsal mechanisms of spatial and serial information in WM. In serial-order rehearsal, which requires remembering the correct sequence of items, the memory of the serial order of each single item is strictly linked with the memory of the other elements in the sequence. In this context, a constant attentional control of item order during rehearsal could be crucial. By contrast, in spatial rehearsal, in which the location of each single item is not linked with the memory of other items’ location, maintenance of item location in space could be configurational and not sequential. In this context, a constant attentional control could be less critical for spatial rehearsal, and the encoding of a concurrent feature can be attained without weakening spatial processing.

To discuss our results in the framework of the working memory model (Baddeley 2000), we could speculate that the phonological loop, crucial for serial-order maintenance, is particularly susceptible to the attentional interference caused by concurrent information whereas the spatial module of auditory working memory appears less prone to interference. Furthermore, several models have been proposed to describe the mechanisms of serial-order processing in humans (see Henson 2001 for a review). For modeling serial-order processing, the analysis of error type during the sequence recall is crucial. In future research on spatiotemporal integration in working memory, it could be interesting to investigate whether the manipulation of attention modulates not only the amount, but also the position and the type of error in serial-order recall and to verify which models fit better with the data.

Finally, as we did not include completely unexpected conditions in our experiments, this model does not predict what would happen in conditions where no attention at all is allocated on the to-be-recalled dimension. It would be interesting for future research to verify whether the recall of spatial and/or temporal features would still be above-chance level in surprise tasks, where the feature to be recalled is fully unexpected during encoding. If so, such a finding would provide strong evidence for automatic encoding of the spatial and/or the temporal dimensions.

To conclude, when keeping track of *where* and *when* auditory events happened in our surroundings, we do not necessarily remember such dimensions in association. Our results suggest that in order to remember spatiotemporal attributes of sounds, we have to pay attention to *when* and/or to *where* sounds are presented. Importantly, attention in encoding is more crucial for remembering when a sound is presented than for remembering where a sound comes from.
